# Supplementary material for: The Properties of Proinflammatory Ly6Chi Monocytes Are Differentially Shaped by Parasitic and Bacterial Liver Infections
Source: Cells. 2022 Aug 16;11(16):2539. doi: 10.3390/cells11162539 (PMC9406626; doi:10.3390/cells11162539)
Supplement: Supplementary file 1 [file cells-11-02539-s001.zip › cells-1861749-supplementary.pdf]

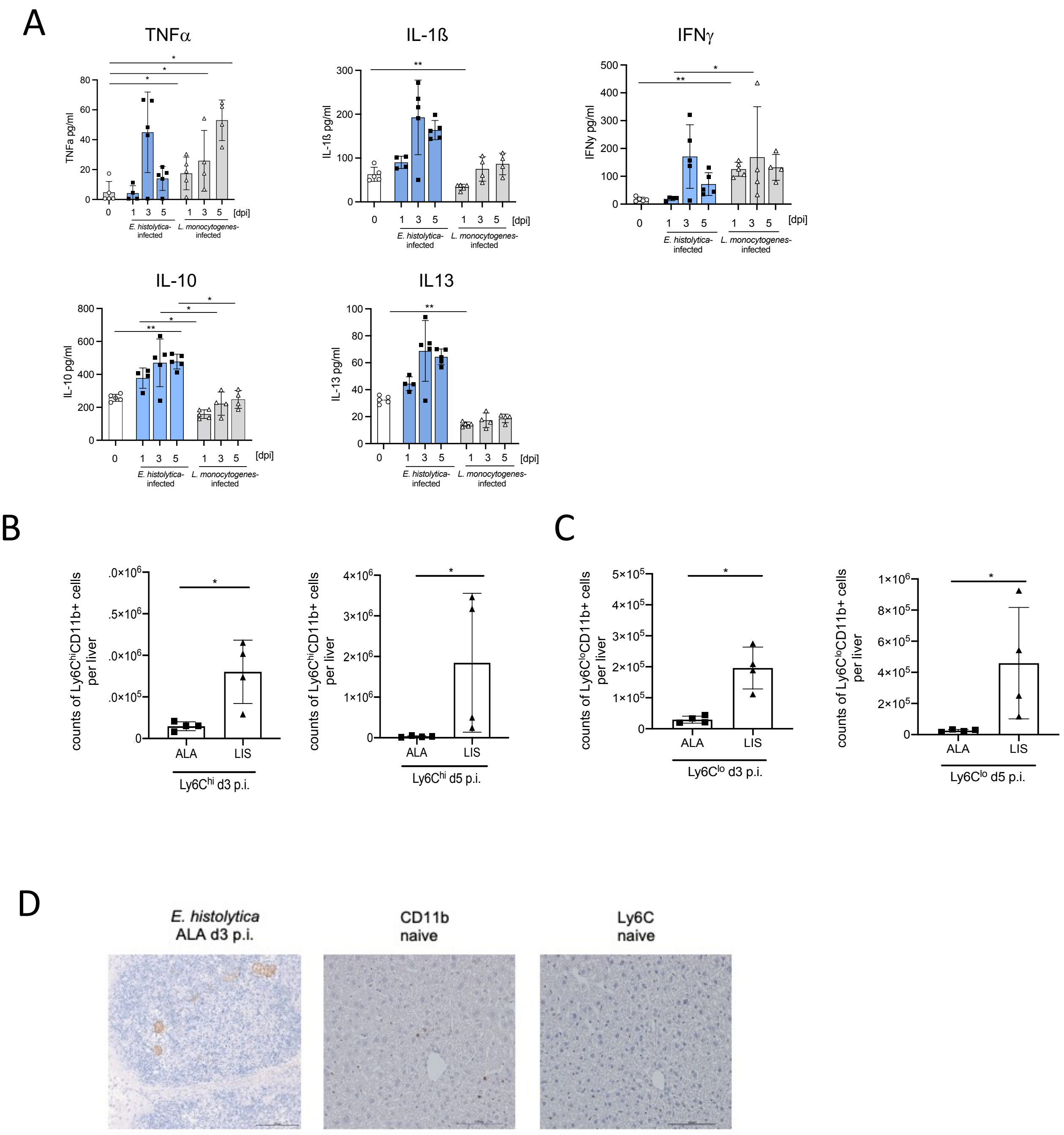

Figure S1: Cytokine milieu during hepatic amebiasis and listeriosis

A) Cytokine concentration in murine liver lysate at indicated time points after intrahepatic infection of $2.5x10^5$ *E. histolytica* trophozoites or intravenous infection of $2x10^4$ *L. monocytogenes* determined by multiplex cytokine assay (LegendPlex, BL). Absolut numbers of pro-inflammatory and anti-inflammatory monocytes on d3 and d5 p.i. from B) *E. histolytica* and C) *L. monocytogenes infected mice*. D) Liver sections from *E. histolytica* infected (d3 p.i.) and naive mice were stained with anti-*E. histolytica* (pool: anti-170kDa Lectin, anti-220kDa Lectin, anti-SOD, anti-peroxiredoxin) and mAbs against CD11b (EPR1344) and Ly6C (ER-MP20). One representative experiment out of 3 (A-D) is shown. Data are expressed as mean ± SEM. p values were determined using the Mann-Whitney U test (*p <0.05; **p <0.01; ***p <0.001).

A

Gene	MeanCount ALA Ly6Chi	MeanCount LIS Ly6Chi	Log2 FoldChange	padj
IIGP1	249,227	24161,955	-6,589112667	0
GM4951	49,9258748	5590,44	-6,817560811	0
KLHL6	997,7453251	5367,57875	-2,428462923	9,464E-267
F830016B08RIK	8,233481227	1208,64125	-7,208449195	1,473E-240
IL18BP	119,02244	2709,64875	-4,524461161	2,809E-228
PLA2G16	138,9782702	1722,8375	-3,651858069	5,373E-225
LY6A	414,3645471	25891,21625	-5,933574663	5,992E-218
WARS	927,3850463	3531,69375	-1,927610345	7,208E-186
AW112010	790,5120389	17607,38875	-4,458975972	5,661E-184
GBP2	2285,971974	29539,3175	-3,706494048	2,123E-176
LAP3	245,9667124	2597,11125	-3,401025731	9,476E-164
GBP6	27,67415277	1027,035	-5,216537222	1,751E-151
GM4841	4,55834516	598,79	-7,07252909	7,654E-126
GLRX	1286,863168	6126,465	-2,247512811	4,659E-119
PSMB9	2167,861175	7670,07125	-1,822573381	4,827E-114
TGTP1	129,736205	4691,92	-5,236978445	7,167E-112
SERPINA3F	103,6206307	2881,6275	-4,840477368	4,521E-106
UPP1	115,1120938	3017,54125	-4,714344332	1,297E-104
IGTP	1690,69752	10332,0175	-2,633640692	6,21E-101
GBP4	170,4038873	4706,25625	-4,875770858	2,262E-100

B

Gene	MeanCount ALA Ly6Clo	MeanCount LIS Ly6Clo	Log2 FoldChange	padj
GM4951	11,8735096	2208,9975	-7,469879617	7,14853E-22
IL18BP	25,26594539	1073,37875	-5,792058644	2,29229E-13
F830016B08RIK	2,816183461	350,4975	-7,701683035	2,67829E-12
IIGP1	140,4051144	8661,08875	-5,76518905	1,10631E-11
PRM1	0	54,20125	-7,000417519	2,01085E-10
SPP1	177,1890049	2607,13625	-4,191041868	1,48013E-09
CD209A	4094,22731	100,5325	5,284376598	4,10385E-09
UPP1	24,8934124	1142,89875	-6,293533846	8,00831E-09
HDC	64,10710943	1675,52875	-6,319989255	1,34919E-08
AA467197	17,41170357	310,53625	-4,961178145	3,00715E-08
GBP2	1074,601145	12786,76625	-3,761186255	3,03927E-08
CXCR2	24,45829754	686,3675	-6,361284538	4,98776E-08
PRSS16	19,41762795	97,12	-3,658139008	6,26113E-08
ASNS	49,80068103	300,66	-2,882330321	1,19752E-07
IL10	3,68206119	323,1175	-6,545475529	2,26944E-07
P2RX3	1,006602645	72,13875	-6,183108842	3,7849E-07
IL1R2	126,1450712	2666,46	-5,762673771	5,71717E-07
MS4A2	6,502531354	271,4125	-6,082275639	6,43744E-07
SULT2B1	16,0341714	327,04375	-4,426278772	1,36361E-06
PRSS34	14,34053248	4969,3575	-6,279807693	3,16683E-06

C

GO-Term number	Enrichment FDR	Genes in list	Functional category
0006955	1,5E-59	925	Immune system process
0044267	2.8E-53	1659	Cellular protein metabolic process
0006950	4,1E-52	1269	Response to stress
0002682	2,7E-44	553	Regulation of immune system process
0002684	8,1E-37	406	Positive regulation of immune system process
0006955	3,3E-36	558	Immune response
0009056	1,6E-35	828	Catabolic process
0044248	4,1E-35	747	Cellular catabolic process
0006464	3,4E-34	1265	Cellular Protein modification process
0036211	3,4E-34	1265	Protein modification process
0080134	3,4E-34	525	Regulation of response to stress
0034097	1,4E-33	402	Response to cytokine
0070887	2,5E-33	1013	Cellular response to chemical stimulus
0043412	5,0E-33	1309	Macromolecule modification
0009893	2,6E-31	1173	Positive regulation of metabolic process
0006952	3,2E-31	564	Defense response
0001775	5,8E-31	393	Cell activation
0031347	8,7E-31	288	Regulation of defense response
0006793	1,5E-30	1058	Phosphorus metabolic process
0071310	3,3E-30	829	Cellular response to organic substance

D

GO-Term number	Enrichment FDR	Genes in list	Functional category
0006952	2,8E-21	67	Defense response
0006955	8,7E-20	82	Immune system process
0009605	2,4E-15	76	Response to external stimulus
0009617	4,5E-15	40	Response to bacterium
0043207	5,8E-15	47	Response to external biotic stimulus
0051707	5,8E-15	47	Response to other organism
0006955	9,9E-15	55	Immune response
0009607	1,6E-14	47	Response to biotic stimulus
0006950	3,2E-14	92	Response to stress
0035456	3,1E-11	13	Response to interferon-beta
0035458	5,7E-11	12	Cellular response to interferon-beta
0070887	3,1E-10	74	Cellular response to chemical stimulus
0034097	3,4E-10	38	Response to cytokine
0006954	5,2E-10	32	Inflammatory response
0071345	6,0E-10	35	Cellular response to cytokine stimulus
0042832	2,5E-09	9	Defense response to protozoan
0071310	4,0E-09	62	Cellular response to organic substance
0001562	4,4E-09	9	Response to protozoan
0002682	7,2E-09	43	Regulation of immune system process
0010033	2,0E-08	74	Response to organic Substance

E

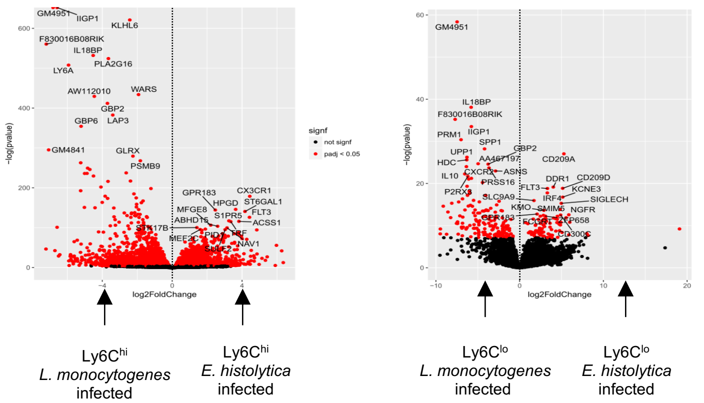

F

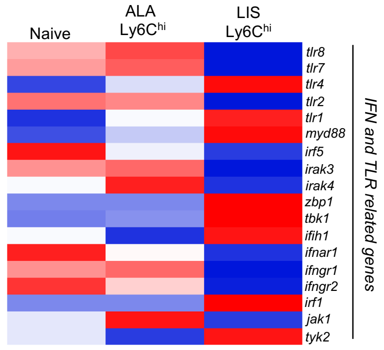

Figure S2: Comparative analysis of transcriptome and FACS data of liver-specific Ly6Chi/lo monocytes from *E. histolytica* and *L. monocytogenes* infected C57BL/6 mice. 20 most significant regulated genes between A) pro-inflammatory and B) anti-inflammatory monocytes. Most abundant and regulated GO-Term (n=20) comparing significant regulated genes C) from pro-inflammatory and D) anti-inflammatory monocytes; E) Volcano-Plot of regulated genes expressed by Ly6Chi and Ly6Clo monocytes. Plotting log-p values against log2-fold changes reveals significantly (red) and non-significantly (black) regulated genes. F) Selection of upregulated IFN and TLR related genes.

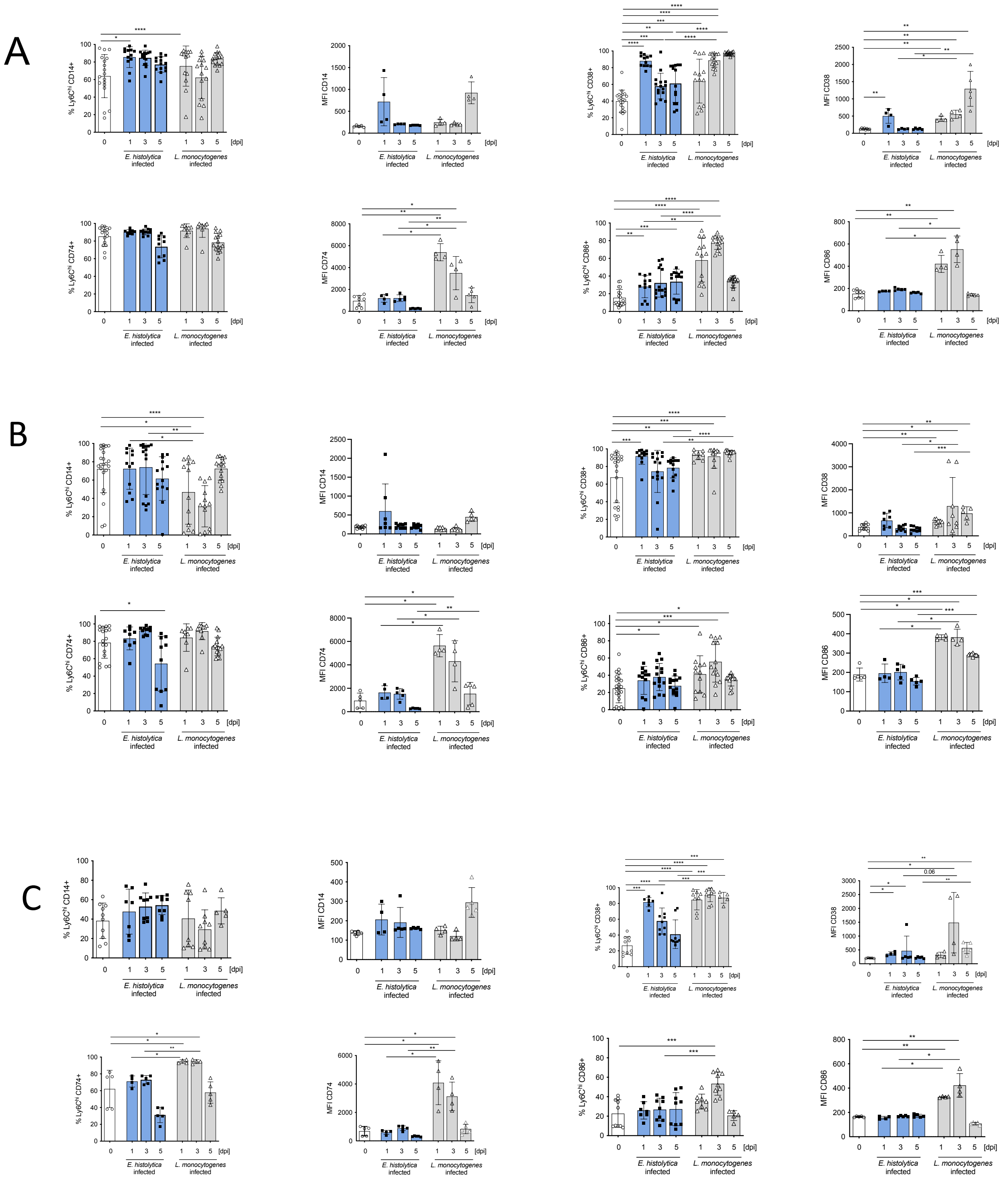

Figure S3: Expression pattern of CD14, CD38, CD74 and CD86 on Ly6C^{hi} monocytes derived from different organs during the course of infection with *E. histolytica* and *L. monocytogenes.*

Determination of percentages of positive cells and MFI for selected markers on Ly6C^{hi} monocytes from A) spleen, B) blood and C) bone marrow. Data were pooled from three independent experiments, MFI data in A-C represent one out of three experiments. Data are expressed as the mean ± SEM. p values were determined using the Mann-Whitney U test (*p <0.05; **p <0.01; ***p <0.001).
